# Review of the Etiopathogenesis and Management Options of Chondrodermatitis Nodularis Chronica Helicis

**DOI:** 10.7759/cureus.2367

**Published:** 2018-03-26

**Authors:** Haneen Salah, Brittany Urso, Amor Khachemoune

**Affiliations:** 1 Medical Student, Alfaisal University College of Medicine; 2 Medical Student, University of Central Florida College of Medicine; 3 Dermatology, State University of New York

**Keywords:** chondrodermatitis nodularis chronica helicis, cnch, winkler's nodule

## Abstract

Chondrodermatits nodularis chronica helicis (CNCH), first described by Max Winkler in 1915, presents as a sore nodule on the helix or antihelix of the external ear. In this paper, we review the etiopathogenesis and management options of CNCH. This condition has a multifactorial etiology; however, sustained pressure from sleeping on one side is the favored theory. Currently, there are many surgical and non-surgical methods of treating CNCH. Most practitioners recommend conservative measures first in their patients, such as pressure-relieving prostheses, prior to surgical treatment. Surgery is the gold standard of therapy with cartilage and wedge excisions yielding recurrence rates of about 10%. Carbon dioxide laser and photodynamic therapy are newer treatment modalities for CNCH, yet they have recurrence rates similar to conservative therapy. In conclusion, due to the high rates of CNCH recurrence, wedge resection is the suggested treatment for CNCH after conservative measures fail.

## Introduction and background

Historical perspective: Chondrodermatitis nodularis chronica helicis (CNCH), also known as chondrodermatitis nodularis helicis (CNH), ear pressure sore, painful nodule of the ear, Winkler’s nodule, or Winkler’s disease, was first described by the dermatologist Max Winkler in 1915, who reported it on eight men presenting with painful nodules on the helix of the external ear in a paper titled Knötchenförmige Erkrankung am Helix (Chondrodermatitis Nodularis Chronica Helicis) [1-3]. In 1918, Foerster reported an additional four cases of CNCH and further defined the clinical, microscopic, and treatment options of CNCH in an additional eight cases in 1925 [2-3].

**Figure 1:**
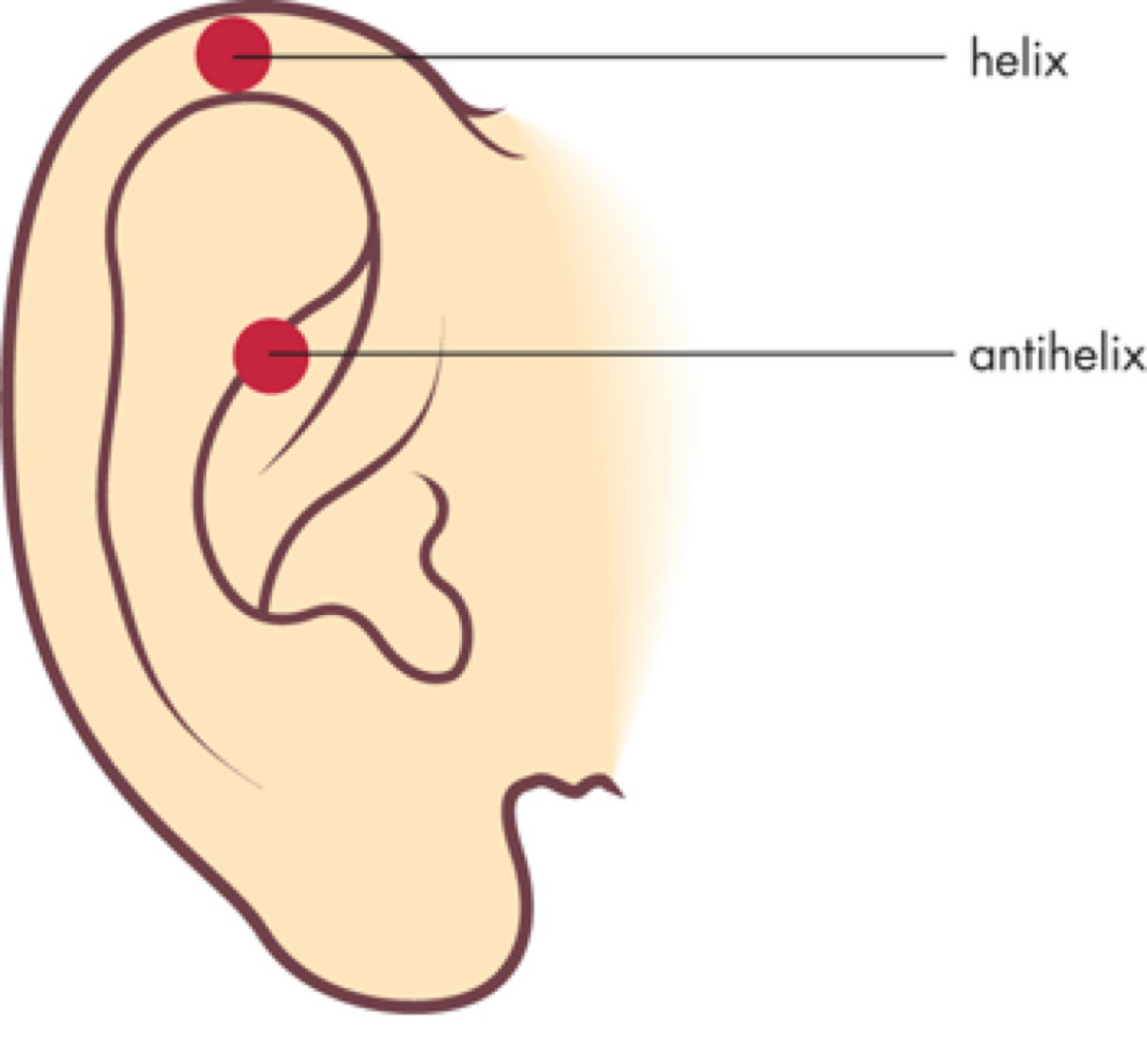
Basic anatomy of the ear The helix and antihelix are the most common locations of chondrodermatitis nodularis chronica helicis (CNCH). Photo credit: John Murtagh's General Practice, 6e. McGraw-Hill Education, 2015.

Clinical presentation: CNCH often presents as a benign painful erythematous nodule fixed to the cartilage of the helix or antihelix of the external ear (Figure 1) [3-6].

**Figure 2:**
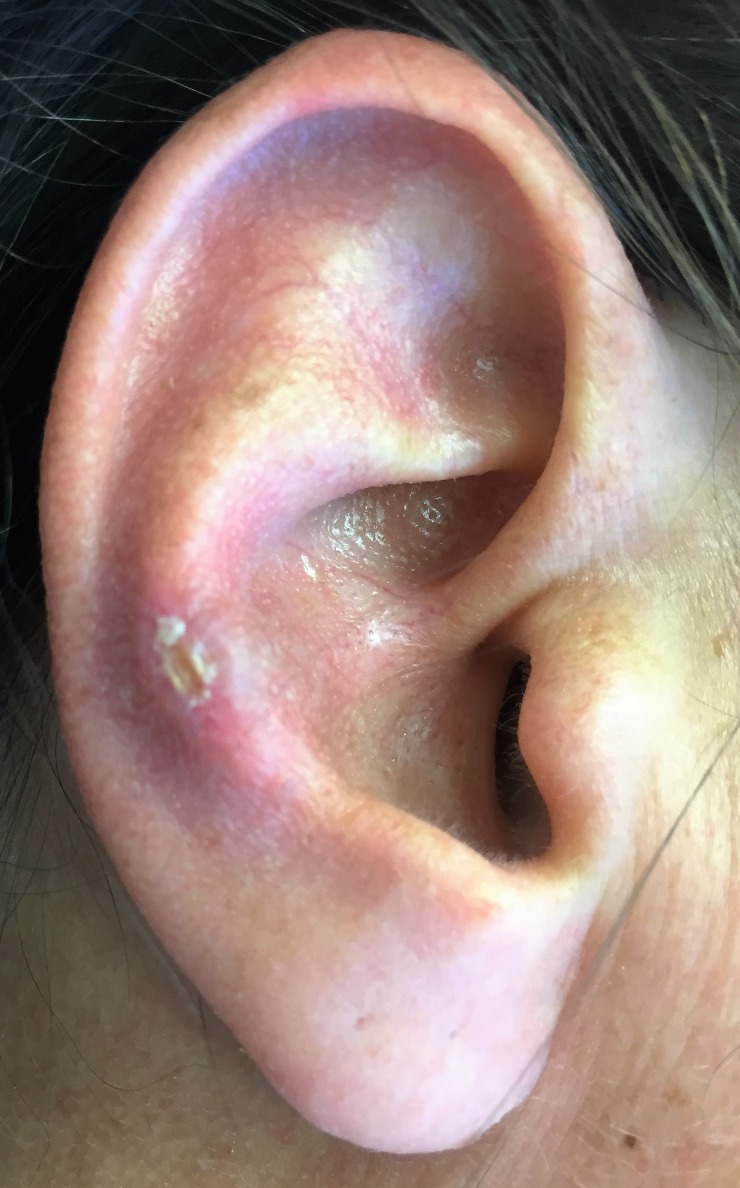
Clinical image of CNCH on the right antihelix CNCH: chondrodermatitis nodularis chronica helicis

The nodules attain their maximum size of 4 mm to 6 mm in a few months and remain unchanged if left untreated [3-5]. Over a period of a few weeks, the nodule develops a central crater, which contains crust-like material [3]. It has been reported that right-sided lesions are more common than left-sided ones. CNCH most commonly occurs on the helix of the external ear in men and on the antihelix in women (Figure 2) [7].

Some authors suggest naming this condition as chondrodermatitis nodularis auricularis due to its location [4]. The unusual presentation of Winkler’s disease was reported as a large nodular growth arising from the tragus, nearly occluding the external auditory canal with a size of 1.5 cm by 2 cm [8].

Epidemiology: CNCH is fairly uncommon; however, it has a predilection for fair-skinned males, aged 40 to 80 years old [5,9]. Though CNCH does affect women, the male to female ratio has been reported to be 10 to 1 [4,10-11]. Typically, CNCH occurs unilaterally; however, bilateral lesions have been reported with an incidence of 3% to 7% [4,10]. Bilateral lesions tend to appear in patients who change their sleeping side after developing CNCH on the contralateral ear [12]. Additionally, CNCH can occur in any age group, with reported cases in children and teenagers [13-14].

## Review

Etiopathogenesis

The etiology of CNCH is unclear. However, it is believed that several factors may contribute to the development of CNCH, and the onset is thought to be related to microtrauma, prolonged excessive pressure, or ischemia to the dermis [15]. The possible contributory factors are as follows:

Age: Aging is another factor that causes the thinning of skin and cartilage, the loss of elastic tissue, and the degeneration of vascular and connective tissue [16]. CNCH symptoms may start because the cartilage becomes less flexible with age, which makes it more vulnerable to pressure damage. Furthermore, older people usually tend to move less while sleeping, which further increases pressure on the ear [12].

Cartilage degeneration: Winkler believed that CNCH occurs secondary to cartilage degenerative changes, which act as an inflammatory stimulus to the skin [17]. Also, degenerative changes in the cartilage and its overlying skin, as a result of pressure necrosis, are thought to be the most common etiological factor [12]. The underlying cartilage may show degenerative changes or necrosis due to direct involvement in the ulcer [18]. One study re-reported the perichondrial vasculitis theory, which was first described by Halter in 1936, in which they observed arteriolar narrowing in 16 patients in the perichondrium in the pinna most remote from the arterial supply, which leads to ischemic changes and death of cartilage [18].

Ear anatomy: The anatomical features of the pinna act as a predisposing factor due to the little subcutis and due to the small blood vessels that supply the skin and the cartilage [8,16,19]. The anatomic features of the ear interfere with adequate healing and lead to secondary perichondritis [16]. The skin of the external ear is tightly stretched over the underlying cartilage, and the circulation in that area is poor due to little subcutaneous tissue [12,16]. Furthermore, the architecture of the ear facilitates the development of CNCH on the most protuberant parts, which are the helix in men, and the antihelix in women [12]. It is also possible that women develop antihelical lesions more commonly because their longer hair shields the helix but not the antihelix [20].

Genetics: Chan et al. in 2008 reported 46-year-old monozygotic male twins who developed CNCH lesions only 36 days apart [17]. One twin presented first with a unilateral lesion, whereas the other twin developed bilateral lesions. This temporal concordance suggests the possibility of but does not prove a partial genetic component in the pathophysiology of CNCH [17].

Glomus-like vascular changes: Small glomus tumors of the helix were identified by Calnan et al. in 1959 when they re-examined the histological features of 21 previously diagnosed cases of CNCH [21]. Three-quarters of these cases revealed unusual epithelioid proliferation in the walls of arteriovenous anastomoses [21]. Similarly to the glomus tumor, CNCH causes pain secondary to both pressure and changing temperature [21-22].

Perforating dermatoses and transepidermal elimination: There is a general agreement that CNCH is a disorder of transepidermal elimination, in which damaged dermal connective tissue (primarily collagen) is engulfed and eliminated by a hyperplastic epidermis [15,23]. Other perforating dermatoses are elastosis perforans serpiginosa, perforating collagenosis, perforating folliculitis, and Kyrle’s disease [24]. In a study that examined 57 lesions from 45 patients, the absence of chondritis/perichondritis in histological examinations supports that CNCH is primarily of a dermal/epidermal origin rather than being a cartilaginous disorder, and a number of its histological features are common to several other perforating dermatoses [22]. Thus, it is suggested that CNCH should be a component of this group of disorders [15].

Pressure: CNCH is more common in people who sleep predominantly on one side, though cases associated with headgear and hearing aids have been reported [10,15,20]. The pressure on the ear likely impedes the blood supply, causing collagen and cartilage damage near the dermis [3,15,25-26]. It has been reported that 77%-99% of the patients with CNCH sleep on the same side as their lesion [5,12,15]. A prolonged period of sustained pressure may expose the underlying cartilage and its perichondrium to ischemia and this may explain why patients usually complain of waking with pain on the side they slept on [23].

Systemic associations: Although CNCH usually presents as an idiopathic lesion, it may occasionally be associated with autoimmune or connective tissue disorders, such as autoimmune thyroiditis, lupus erythematosus, dermatomyositis, and scleroderma [27]. These associations may be more common in pediatric and female groups [27]. An autoimmune workup should be performed if the patient presents with CNCH at a younger age or if it is suspected from patient history [28]. One study correlated CNCH with cardiovascular health in 17 patients and found that 15 of those patients (88.2%) had high cholesterol and lipid levels. This suggests that defective blood supply to the pinna may have a role in CNCH [14].

Trauma: A history of mechanical injury is an important cause and should be addressed in each case [15]. Repetitive minor traumas to the helix of the ear cause chronic inflammation of the cutis and perichondrium, which can progress to vascular failure [17,25]. It is believed that CNCH is a result of repeated trauma, leading to ischemic changes of the skin and cartilage [16]. Furthermore, minor trauma from headgear or telephone headsets, nun’s coifs, hats, ear piercings, and Bluetooth ear devices could cause CNCH [29].

Management options

The spontaneous resolution of CNCH has been reported in some studies; however, it is not the rule or always the case, and these lesions often require treatment [15,30]. A shave biopsy of the lesion is recommended to diagnose the lesion and rule out malignancy [12-13]. Nonsurgical procedures have a greater chance of recurrence when compared to surgical procedures [17,25]. Recurrences often interfere with the treatment if all lesion sites are not eradicated [8]. Overall, management can be challenging and recurrence is common [20]. The patients usually seek medical help when the pain interferes with their sleep [8]. CNCH can be managed by the following:

A. Non-Invasive Methods

Carbon dioxide laser (CO_2_ laser): The CO_2_ laser vaporizes the nodules, as well as the underlying cartilage. One study reports a 92% cure rate in a study of 12 patients over a nine-month period [3]. There were no infection complications after laser treatment, and cosmetic results were excellent after three to four weeks of treatment [31].

Injectable collagen implants: Occasionally, injectable collagen implants, cushions between the skin and cartilage, have been used for conservative treatment [15,25,30,32]. One study reported complete symptomatic relief in five patients after collagen implants, with no recurrence in a 16 months' period [33].

Intralesional steroid injections: Intralesional steroid injections have been reported to be used in treating CNCH [15,34-36]. Treatment includes intralesional corticosteroid (triamcinolone acetonide) with a success rate of 33% [5,15,25,36]. Although corticosteroid injections are sometimes successful as an initial treatment, a large majority of the patients require further treatment [12]. The use of intralesional steroids in cohorts of patients showed an overall response rate of up to 40% [23].

Nitroglycerin gel: Another conservative measure is nitroglycerin gel. This acts to vasodilate arteriolar smooth muscles and is theorized to help reverse necrosis of cartilage. There was a 92% cure rate among 12 CNCH patients who used 2% nitroglycerin gel twice daily for three months [37]. Another study used 0.2% topical nitroglycerin and reported 17 of 29 (58%) patients with complete resolution of the CNCH over a roughly two-month period without a headache, which is a rarely reported symptom of 2% topical nitroglycerin [38].

Photodynamic therapy (PDT): This method uses a light source to temporarily improve blood flow to the lesion and produce a cytotoxic effect toward the lesion pretreated with a photosensitizing agent, such as 20% 5-aminolevulinic acid cream or 16% methyl aminolevulinate (MAL) [3,34,39]. In a study of five recalcitrant nodules, there was an 80% cure rate using 20% 5-aminolevulinic acid cream with PDT [3]. Other studies support an 80%-100% cure rate; however, sample sizes for these studies are small [34]. In a study using MAL, 43 patients underwent one to six PDT sessions, with each session spaced 15 to 30 days apart [39]. Two patients discontinued treatment [39]. Thirty-three patients out of the 41 patients in the study had a complete response to treatment. Five patients had a partial response and three patients had no response [39]. Recurrence occurred in 10 of 41 (24.4%) cases [39].

**Figure 3:**
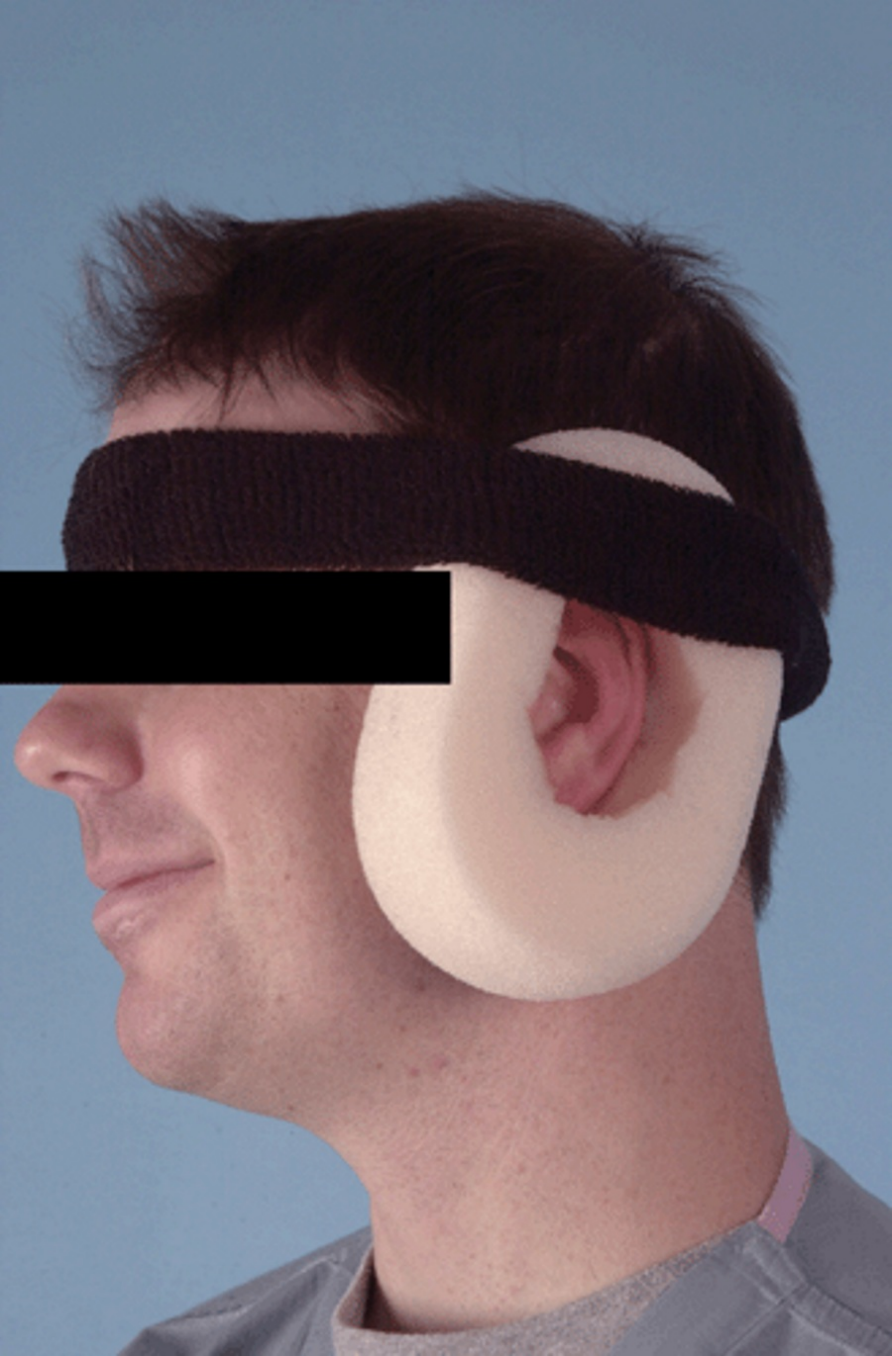
Homemade pressure-relieving device

Removal of causative factor/relieving pressure: The elimination/avoidance of triggering factors, such as pressure, trauma, or actinic (solar) damage were suggested as the first-line modality in treating CNCH patients [11,15,23]. The required treatment starts with preventing the patient from sleeping on the affected ear [40]. Moncrieff et al. reported a study in which patients were given the option of undergoing surgical treatment or treatment with a homemade, pressure-relieving prosthesis [23]. Patients were told to make their prosthetic by encircling the ear with a foam pad and securing it with a padded headband (Figure 3) [23].

During the study, 61 patients underwent surgical treatment. Of those, 41 (67%) were included in the study. Fifteen patients were managed with the prosthetic [23]. Of the 15 patients who underwent conservative treatment, 13 patients (87%) had resolution at the one-month follow-up and did not require surgical intervention. The authors of this study recommend the use of a homemade prosthetic for CNCH for a one-month trial prior to undergoing surgical management [23]. Prosthetics are recommended as a cost-effective way of treating CNCH with few adverse effects; however, neck pain and stiffness have discouraged some patients from using it [5]. Patient compliance often limits recovery, so foam bandages, which are easily transported and less bulky are other options [11,34].

Topical steroids: Topical steroids have been used in a small cohort study of five patients with a 100% success rate [23,41]. If the lesion is ulcerated, topical steroids can be applied with a light dressing [34]. A success rate of 27% has been reported when using topical and intralesional steroid concurrently [5].

B. Invasive Methods

**Figure 4:**
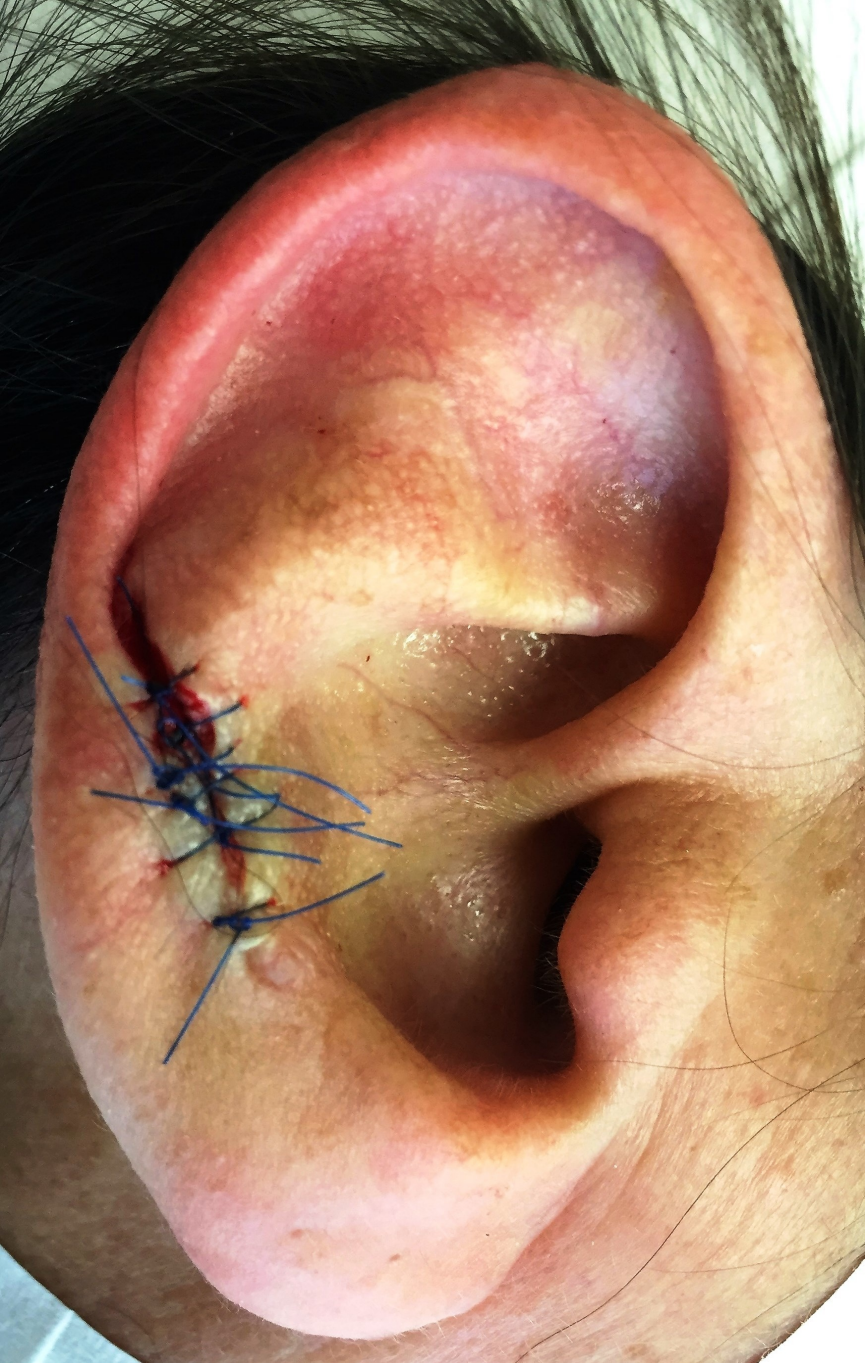
Clinical image of CNCH status post wedge resection CNCH: chondrodermatitis nodularis chronica helicis

Cartilage excision: Based on the theory that CNCH is caused by pressure necrosis of the protuberant cartilage, excellent cosmetic results and a low recurrence rate of about 10% were found when using a cartilage smoothing procedure that was performed on 34 patients. This technique involves the removal of the underlying protuberant cartilage only while maintaining the overlying skin [8,12,30]. The excision of the cartilage alone has been documented to be therapeutically and cosmetically effective to simplify the surgical procedure (Figure 4) [15].

A novel excision and reconstruction technique has been reported for both helical and antihelical lesions. This procedure involves the removal of the lesion and the overlying skin, smoothing the underlying cartilage to prevent recurrence, and using advancement flaps to cover the dissected area [9].

Curettage: The curettage procedure is completed using sharp curettes [30]. The necrotic cartilage in CNCH is soft and removed easily, and the endpoint is reached when the curette is repelled by a firm elastic cartilage [8,15,30]. However, a recurrence of 31% has been reported after using this technique [42].

Wedge excision: Surgery is still the gold standard therapy for CNCH [5,8,17,23,25]. Treatment is surgical removal by the local excision of the lesion, including a small wedge of underlying cartilage and reconstructing the skin and cartilage at the margins [32]. One study conducted on 55 patients has reported that surgical excision is the treatment of choice, but the rate of recurrence is high (11%) [43]. Good results have been reported with wedge excision; however, recurrence up to 10% usually happens at the margins of the excised cartilage [12,15]. Furthermore, repetitive wedge excision after recurrence may yield a deformed and asymmetrical ear [12,15]. The recurrence rate of CNCH is high unless the focus of the damaged cartilage is removed [34]. 

Table 1 shows a summary of non-invasive and invasive management modalities, including sample size, available success/cure rate, recurrence rate, advantages, and disadvantages:

**Table 1:** Summary table NR = not reported

Method	Sample size (patients)	Success/cure rate	Recurrence rate	Advantages	Disadvantages	Reference
A- Non-Invasive methods						
Carbon dioxide laser (CO2 laser)	12	NR	No recurrences after 2-15 months	Immediate pain relief following laser surgery Healing with excellent cosmetic results within 3- 4 weeks	None	31
Injectable collagen implants	5	100%	None in 16 months since first treatment	Corrects the deformity contour	None	33
Intralesional steroid injections	60	33%	NR	2/3 of patients get initial benefit within 1-2 days after a single injection	Low cure rate	36
Nitroglycerin gel	29	93%	NR	Improves appearance	Headache in one case	38
Photodynamic therapy (PDT)	43	76.7%	23.3%	Pain relief	NR	39
Removal of causative factor/relieving pressure (Doughnut pillow)	15	87%	NR	Comfortable to wear over prolonged periods Cost effective	Two patients had no improvement due to low compliance	23
Topical steroids	5	100%	NR	NR	NR	41
B- Invasive Modalities						
Cartilage excision	34 patients/37 lesions	NR	NR	Safe and simple	One patient required revision surgery	12
Curettage	142	NR	31%	Satisfactory from a cosmetic point Equal to more invasive procedures	NR	42
Wedge excision	55 patients/62 lesions	89%-96%	11%	NR	NR	43

## Conclusions

In summary, CNCH is a multifactorial condition with several potential etiologies. The most likely cause of CNCH is ischemia or microtrauma related to sleeping on one side. Many treatment methodologies are emerging, including surgical and non-surgical; however, the first-line treatment is to relieve pressure from the site through the use of a donut pillow or a homemade pressure-relieving device. Intralesional steroids are often second-line; however, CNCH reoccurrence is common. Other topical treatments, such as topical nitroglycerin, show promising results, but further studies with large sample sizes need to be completed. The gold standard of therapy is surgical excision via wedge resection, despite reoccurrence if the defective cartilage is not removed.

## References

[1] Wagner G, Liefeith J, Sachse MM (2011). Clinical appearance, differential diagnoses and therapeutical options of chondrodermatitis nodularis chronica helicis Winkler. J Dtsch Dermatol Ges.

[2] Winkler M (1915). Chondrodermatitis nodularis chronica helicis [Article in German]. Arch f Dermat.

[3] Kechichian E, Jabbour S, Haber R, Abdelmassih Y, Tomb R (2016). Management of chondrodermatitis nodularis helicis: a systematic review and treatment algorithm. Dermatol Surg.

[4] Juul Nielsen L, Holkmann Olsen C, Lock-Andersen J (2016). Therapeutic options of chondrodermatitis nodularis helicis. Plast Surg Int.

[5] Sanu A, Koppana R, Snow DG (2007). Management of chondrodermatitis nodularis chronica helicis using a 'doughnut pillow'. J Laryngol Otol.

[6] Murtagh J (2015). John Murtagh's General Practice. http://murtagh.mhmedical.com/book.aspx?bookID=1522.

[7] Khurana U, Solanki LS, Dhingra M (2015). A man with painful nodules on both ears. JAMA Otolaryngol Head Neck Surg.

[8] Nagaraj B, Ravi G, Reddy S (2012). An unusual case of Winkler's disease of pinna. Transl Biomed.

[9] Yaneza M, Sheikh S (2013). Chondrodermatitis nodularis chronica helicis excision and reconstruction. J Laryngol Otol.

[10] Oelzner S, Elsner P (2003). Bilateral chondrodermatitis nodularis chronica helicis on the free border of the helix in a woman. J Am Acad Dermatol.

[11] Travelute CR (2013). Self-adhering foam: a simple method for pressure relief during sleep in patients with chondrodermatitis nodularis helicis. Dermatol Surg.

[12] de Ru JA, Lohuis PJ, Saleh HA, Vuyk HD (2002). Treatment of chondrodermatitis nodularis with removal of the underlying cartilage alone: retrospective analysis of experience in 37 lesions. J Laryngol Otol.

[13] Avitia S, Hamilton JS, Osborne RF (2005). Chondrodermatitis nodularis chronica helicis. Ear Nose Throat J.

[14] Karabulut YY, Senel E, Dölek Y (2013). Frequency and etiology of chondrodermatitis nodularis chronica helicis. Indian J Otol.

[15] Sehgal VN, Singh N (2009). Chondrodermatitis nodularis. Am J Otolaryngol.

[16] Dreiman BB (2007). Chondrodermatitis nodularis chronica helicis treated with antia-buch reconstruction: review and case report. J Oral Maxillofac Surg.

[17] Chan HP, Neuhaus IM, Maibach HI (2009). Chondrodermatitis nodularis chronica helicis in monozygotic twins. Clin Exp Dermatol.

[18] Upile T, Patel NN, Jerjes W, Singh NU, Sandison A, Michaels L (2009). Advances in the understanding of chondrodermatitis nodularis chronica helices: the perichondrial vasculitis theory. Clin Otolaryngol.

[19] Kulendra K, Upile T, Salim F, O’Connor T, Hasnie A, Phillips DE (2014). Long-term recurrence rates following excision and cartilage rim shave of chondrodermatitis nodularis chronica helicis and antihelicis. Clin Otolaryngol.

[20] Kaur RR, Lee AD, Feldman SR (2010). Bilateral chondrodermatitis nodularis chronica helicis on the antihelix in an elderly woman. Int J Dermatol.

[21] Calnan J, Rossatti B (1959). On the histopathology of chondrodermatitis nodularis helicis chronica. J Clin Pathol.

[22] Ramsay H, Garrido M, Smith A (1999). Chondrodermatitis nodularis helicis chronica-a clinico-pathological study. Br J Dermatol.

[23] Moncrieff M, Sassoon EM (2004). Effective treatment of chondrodermatitis nodularis chronica helicis using a conservative approach. Br J Dermatol.

[24] Yoshinaga E, Enomoto U, Fujimoto N, Ohnishi Y, Tajima S, Ishibashi A (2001). A case of chondrodermatitis nodularis chronica helicis with an autoantibody to denatured type II collagen. Acta Derm Venereol.

[25] Senel E (2010). Chondrodermatitis nodularis chronica helicis. Clin Med Insights Dermatol.

[26] Kumar P, Barkat R (2017). Chondrodermatitis nodularis chronica helicis. Indian Dermatol Online J.

[27] Sifuentes Giraldo WA, González-García C, de las Heras Alonso E, de la Puente Bujidos C (2014). Chondrodermatitis nodularis chronica helicis in a patient with systemic sclerosis associated with primary biliary cirrhosis (Reynolds syndrome): a case report. Eur J Rheumatol.

[28] Grippe K (2017). Painful, nonbleeding lesion on the ear. Clinical Advisor.

[29] Ortiz A, Martin P, Dominguez J, Conejo-Mir J (2015). Cell phone-induced chondrodermatitis nodularis antihelicis. Actas Dermosifiliogr.

[30] Naqash MM, Salati SA (2013). Chondrodermatitis nodularis chronica helicis — a review. J Pak Assoc Derma.

[31] Taylor MB (1991). Chondrodermatitis nodularis chronica helicis. Successful treatment with the carbon dioxide laser. J Dermatol Surg Oncol.

[32] Jacob KJ, Satheesh S, Menon P, Saju KG (2005). Winkler's disease. Indian J Otolaryngol Head Neck Surg.

[33] Greenbaum SS (1991). The treatment of chondrodermatitis nodularis chronica helicis with injectable collagen. Int J Dermatol.

[34] Shah S, Fiala KH (2017). Chondrodermatitis nodularis helicis: a review of current therapies. Dermatol Ther.

[35] Thompson LD (2007). Chondrodermatitis nodularis helicis. Ear Nose Throat J.

[36] Cox NH, Denham PF (2002). Intralesional triamcinolone for chondrodermatitis nodularis: a follow‐up study of 60 patients. Br J Dermatol.

[37] Yélamos O, Dalmau J, Puig L (2013). Chondrodermatitis nodularis helicis: successful treatment with 2% nitroglycerin gel. Actas Dermosifiliogr.

[38] Sanz-Motilva V, Martorell-Calatayud A, Gutierrez Garcia-Rodrigo C (2015). The usefulness of 0.2% topical nitroglycerin for chondrodermatitis nodularis helicis [Article in Spanish]. Actas Dermosifiliogr.

[39] García-Malinis AJ, Turrión-Merino L, Pérez-García B, Saceda-Corralo D, Harto-Castaño A, Gilaberte Y (2017). Observational study of chondrodermatitis nodularis helicis treated with methyl aminolevulinate photodynamic therapy. J Am Acad Dermatol.

[40] Ali FR, Healy C, Mallipeddi R (2016). Hemorrhoid cushions for chondrodermatitis nodularis helicis (CNH): piling off the pressure. J Am Acad Dermatol.

[41] Beck MH (1985). Treatment of chondrodermatitis nodularis helicis and conventional wisdom?. Br J Dermatol.

[42] Kromann N, Høyer H, Reymann F (1983). Chondrodermatitis nodularis chronica helicis treated with curettage and electrocauterization: follow-up of a 15-year material. Acta Derm Venereol.

[43] Feldman AL, Manstein CH, Manstein ME, Czulewicz A (2009). Chondrodermatitis nodularis auricularis: a new name for an old disease. Plast Reconstr Surg.

